# Magnetic resonance venography for 3-dimensional live guidance during venous sinus stenting

**DOI:** 10.1186/s42155-020-00158-7

**Published:** 2020-09-11

**Authors:** Vaishnavi Kishore, Sri Hari Sundararajan, Raphael Doustaly, Marissa Michael, Dwight Xuan, Thomas Link, Benjamin Rapoport, Athos Patsalides

**Affiliations:** 1 GE Healthcare, 3000 N Grandview Blvd, Waukesha, WI 53188 USA; 2 Department of Neurosurgery, New York Presbyterian Hospital/Weill Cornell Medical Center, 525 East 68th St, New York, NY 10065 USA; 3 Department of Aerospace Medicine, Los Angeles Air Force Base, 483 North Aviation Boulevard, Los Angeles, CA 90245 USA

**Keywords:** Venous sinus interventions, Dural venous sinus stenting, Vessel ASSIST, 3D live guidance, 3D fusion, MRV fusion overlay, IIH, Idiopathic intracranial hypertension, Augmented fluoroscopy

## Abstract

**Purpose:**

The purpose of this study was to report the technique for intraprocedural guidance of endovascular Venous Sinus Stenting procedures using 3-Dimensional (3D) Magnetic Resonance Venography (MRV) as an overlay on live biplanar fluoroscopy.

**Materials and methods:**

Venous sinus stenting procedures performed between April and December, 2017 with 3D MRV fusion for live guidance were reviewed in this study. A thin-slice, contrast-enhanced MR Venogram was used to create 2 3D models – vessels and skull – for procedural guidance via augmented fluoroscopy (Vessel ASSIST, GE Healthcare, Chicago, IL). The skull model was used in the registration of the 3D overlay on both the frontal and lateral planes, which required 1–2 min of procedural time. The vessel model was used to mark landmarks such as the cortical vein ostia and stenosis on the 3D overlay fused with biplanar fluoroscopy.

The retrospective imaging review was conducted by 3 neurointerventionalists and relied on a consensus confidence ranking on a 3-point Likert scale from 1- low confidence to 3- high confidence. The neurointerventionalists first reviewed the conventional 2-dimensional pre-stent deployment fluoroscopy images and then reviewed the corresponding images with the 3D MRV overlay. They ranked their confidence in their understanding of cortical venous anatomy for each group. Statistical analysis was performed using a Paired T Test at a 99% confidence interval.

**Results:**

Ten cases were included in the retrospective image review. Operator confidence regarding the location of cortical veins was significantly increased using 3D MRV fusion during venous sinus stenting procedures (1.9 vs 2.9, *p* = .001).

**Conclusion:**

3-Dimensional MRV fusion is feasible and helpful in understanding the venous sinus anatomy and location of important cortical veins during venous sinus stenting procedures.

## Introduction

Idiopathic Intracranial Hypertension (IIH) is a condition characterized by increased intracranial pressure manifesting with headaches, pulse-synchronous tinnitus, diplopia, and visual field loss from papilledema (Dinkin and Patsalides 2017). Stenosis of the lateral venous sinuses has been associated with IIH, leading to the development of venous sinus stenting as a treatment option in medically refractory IIH. This approach is associated with high rates of technical success, favorable clinical outcomes, and lower complication profile, noting that a comprehensive understanding of the medical and alternative surgical options alongside consistent long-term follow-up intervention is necessary to ensure maintenance of such benefits (Kalyvas et al. 2017).

One technical difficulty of venous sinus stenting procedures arises from the difficulty in obtaining appropriate venous road mapping. As the procedure involves advancing (micro)wires, (micro)catheters, and stents in venous sinuses in a retrograde fashion, there is a risk of inadvertent placement of a wire in a cortical vein resulting in catastrophic hemorrhage (Lavoie et al. 2018). Of course, one way to make catheterization of the venous system safer is obtaining road map via a trans-arterial injection (Daggubati and Liu 2019).

Another potential risk of the procedure is compromised flow of cortical veins draining in the lateral venous sinus in the area of stenosis and stent (Boddu et al. 2016). Knowing the location of the cortical veins and their relation to the stent construct is important in preventing this condition, as we can avoid overlapping stents at the ostium of major cortical veins such as the vein of Labbe. In our institution we explored whether non-invasive imaging can be used intra-procedurally to help avoid the potential pitfalls of retrograde navigation and understand the location of cortical veins. We evaluated whether Magnetic Resonance Venography (MRV) can be further leveraged intra-procedurally by fusing a 3D MRV model of the venous sinuses with biplanar fluoroscopy using Vessel ASSIST (GE Healthcare, Chicago, IL).

The purpose of this study was 1) to report the technique for intraprocedural 3D MRV guidance, and 2) to report our initial experience and clinical potential of this technique.

## Methods

### Patient selection

Patients who underwent venous sinus stenting procedures with a 3D MRV-fusion overlay between April and December of 2017 were included in this study, which was approved by our Institutional Review Board with a waiver of informed written consent. Cases with poor quality pre-procedural MRVs (with motion degradation or inadequate visualization of the sinuses) were excluded. 10 consecutive cases fit these criteria during this time period and were included in the study.

### Procedural stages to create and integrate fusion images

#### Building the 3D MRV model

**Fig. 1:**
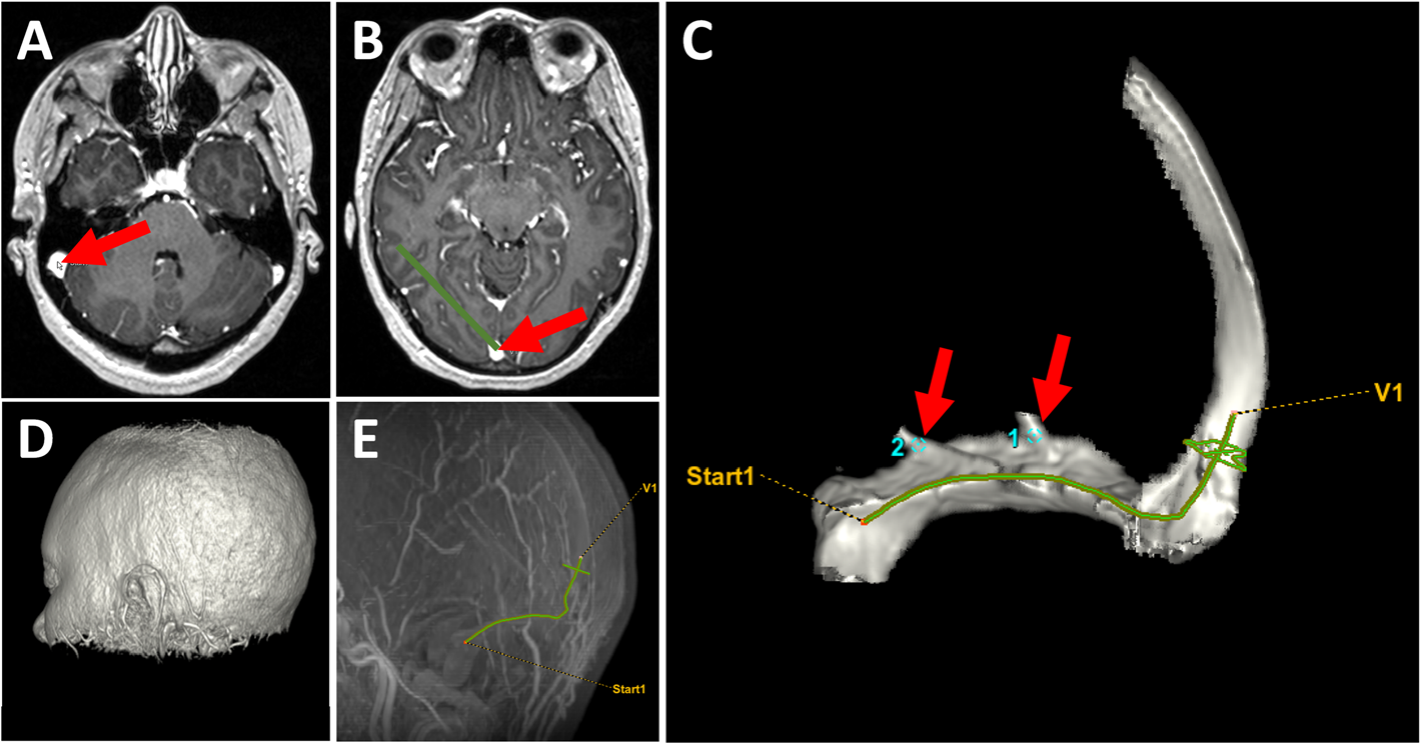
3D Vessel Model. The selection of the start and end points for extraction in the sigmoid sinus and superior sagittal sinus can be seen in **a** and **b**, respectively. The resultant 3D VR vessel model with 2 points of interest (labelled 1 and 2) and the skull model, which is used to register the MRV model with fluoro, are pictured in **c** and **d**. A sagittal 3D MIP of the venous anatomy with the vessel trace highlighted in green and the start and end points defined can be seen in **e**

**Fig. 2:**
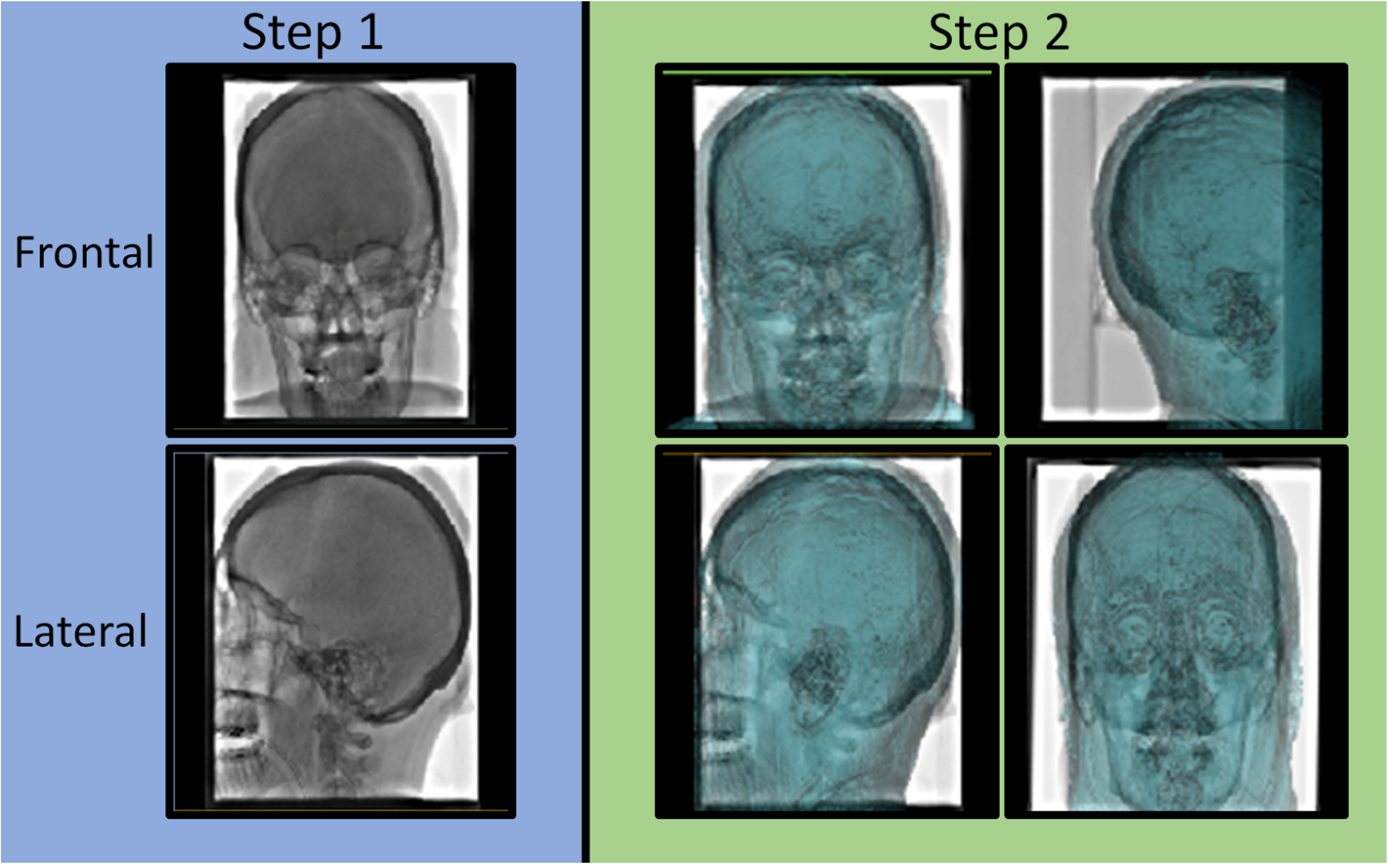
MRV Model Registration Procedure. Step 1 is performed in the angiosuite and requires the operator to acquire two fluoroscopic images at different obliquities in both the frontal and lateral planes. Step 2 is performed in the control room and allows the user to register the acquired images with the 3D MRV skull model in both the frontal and lateral planes

As part of the standard pre-procedural assessment, all patients from the study group had a thin-slice, contrast-enhanced MR Venogram. All institutional MRV exams were performed on 3 Tesla units (Siemens MAGNETOM Skyra and GE Healthcare Signa Architect) with coronal 2D time of flight, sagittal 3D phase contrast (INHANCE/NATIVE), and 3D thin section T1 pre and postcontrast sequences (SPGR or MPRAGE). Contrast enhanced sequences were acquired following weight-based intravenous administration of gadobutrol (Gadavist). The fusion images used for MRV-guidance relied on two 3D models: a vessel model and a skull model. The vessel model was created using vessel segmentation software (Vessel ASSIST, GE Healthcare, Chicago, IL) for 3D extraction of the venous sinuses on a preprocedural MR venography series. The segmentation required the selection of two end points, one in the superior sagittal sinus and one in the sigmoid sinus, on the multi-planar reformatted MRV images (Fig. 1, a-b). The skull model was used to facilitate accurate registration between the pre-procedural MRV models and live fluoroscopy (Figs. 1, d and 2).

#### Registering the 3D MRV model with live fluoroscopy

**Fig. 3:**
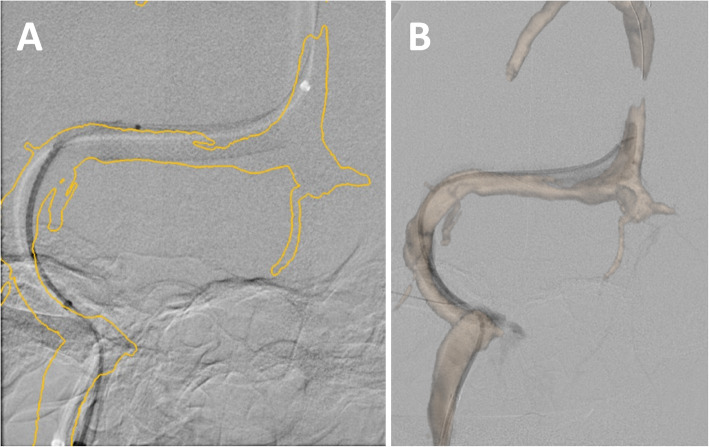
3D MRV model overlaid on fluoroscopy during a Venous Sinus Stenting procedure. The outline and volume rendered views of the overlay can be seen in **a** and **b**, respectively. The slight misregistration can be attributed to device bias of the vessel

Registration between the 3D model and fluoroscopy was performed using a process involving two fluoroscopic acquisitions performed at different obliquities in the frontal and lateral planes. The 3D skull model from the preoperative MRV was aligned with the cranium in each fluoroscopic view (Fig. 3), and the registration was confirmed by imaging the vessels with either an inserted guidewire or a contrast injection. The registered model of the venous sinus was fused with live fluoroscopy so that the operator could visualize the 3D overlay on both the frontal and lateral planes by using the related foot pedal. Landmarks such as Points of Interest that were marked on the 3D model were also displayed on the overlay and were used to denote the location of the cortical veins, stenosis, and other landmarks as necessary.

Registration of the 3D model could be easily refined by the operator during the procedure, if necessary, from the tableside or from the workstation. The opacity and threshold of the 3D model were also adjustable at tableside to allow for better visualization of catheters and wires during the procedure.

The registration required 1–2 min of procedural time and was performed with the patient on the table. The time spent in the registration process mostly consisted of image capture, and the additional time to register the 3D model with the lateral plane as opposed to only the frontal plane was negligible. Building the models and optimizing their appearance for an overlay required approximately 3–5 min by the technologist in the control room without additional impact on procedural time, as the model could be built prior to the case itself.

The 3D model and cortical vein ostia markers were used primarily during stent deployment to optimize the placement of the proximal and distal landing positions of the stent and make sure there was no overlap of the stent with cortical vein ostia as seen in Fig. 3. The overlaid 3D model provided the benefit that it could be seen at all bi-plane angulations without re-registration modification, unlike a 2D roadmap.

### Image review

Images from the 3D MRV overlay cases included in the study were retrospectively assessed by three neuro-interventionalists with 12, 2, and 2 years of experience, who were not involved with the treatments. First, the neuro-interventionalists reviewed the 2D pre-stent deployment images without MRV-fusion from each case to evaluate their confidence levels in their understanding of the cortical venous anatomy by consensus on a 3-point Likert scale, from 1 - low confidence to 3 - high confidence. They then reviewed images with the 3D overlay to evaluate the same and ranked their confidence levels on the same scale. Cases were reviewed in a random order and blinded fashion.

### Statistical analysis

Categorical variables were presented as numbers and percentages and were compared using a Paired T Test at a 99% confidence interval to compare the mean operator confidence levels for the 2 groups. Analyses were conducted using Microsoft® Excel® (Build 11,929.20708) and Minitab® 17 statistical software (2010) (version 17.3.1; Minitab Inc.).

## Results

For comparing conventional images and MRV-guided images from the same procedures, 10 venous sinus stenosis cases with saved images of the 3D overlay on the cortical veins were reviewed to assess their clinical utility. The consensus confidence levels reached by the reviewers are shown in Table 1.

**Table 1:** Operator Confidence Levels in 3D Overlay versus 2D Conventional Imaging

	2D Conventional Imaging Operator Confidence	3D Overlay Imaging Operator Confidence	*p*-value
Cortical Venous Anatomy	1.90 +/− 0.54	2.90 +/− 0.30	0.001

**Fig. 4:**
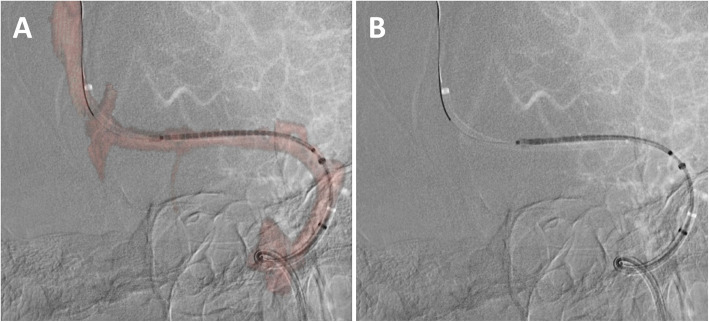
Comparison of 3D MRV Fusion imaging (**a**) and 2D Conventional imaging (**b**) prior to stent deployment

Operator confidence was significantly increased regarding the location of major cortical veins using 3D MRV-fusion during the venous sinus stenting procedures (1.9 vs 2.9, *p* = .001). A comparison of the imaging from both methods is shown in Fig. 4.

## Discussion

In this analysis, we show that MRV overlay for venous sinus stenting is feasible and helpful in understanding the venous sinus anatomy and location of important cortical veins during the procedure. Registration of the MRV models with the 2D images required minimal extra time for the operators and enabled the physicians to better understand the venous anatomy during the procedure, which in turn allowed for safer catheterization of the venous sinuses without the need for arterial catheterization and injection for road map. This can be especially useful for less experienced users and in cases of complex anatomy. Understanding the location of cortical venous drainage with respect to the transverse and sigmoid sinuses allows the operator to avoid overlapping stents across the ostia of such veins and therefore decreases the chance of impairment of cortical venous drainage from excessive metal coverage, a known risk of the procedure (Boddu et al. 2016).

A similar study was performed with an analogous vessel navigation software that provided venous guidance as an overlay on only a single plane of imaging and showed that it was safe and feasible (Blanc et al. 2018). In contrast, this study assessed the clinical utility of a 3D overlay on both (Frontal and Lateral) imaging planes used during the procedures with negligible extra time to provide fusion imaging on the lateral vs frontal plane only.

CT modalities have also been used in diagnosing the presence of dural venous sinus stenosis or occlusions, noting that they are either performed in more emergent settings or in patients with MR incompatible devices (such as pacemakers). Although osseous landmarks could be registered with greater accuracy on the CT modality relative to MRI, there are noteworthy limitations with the use of thin section CT venography compared to MR venography. The relatively larger contrast volumes needed in performing CT venography poses a risk for patients with reduced Glomerular Filtration Rates (GFRs), noting that patients with GFR less than 60 are at risk of further contrast-induced nephropathies compared to the average population. This is in contradistinction to the low-volumes of gadolinium-based agents used in MR venographic examinations, noting the less that 0.07% chance in development of nephrogenic systemic fibrosis in patients with markedly reduced GFRs less than 30 (Woolen et al. 2019). Radiation doses of gated CT venography exams are also a significant factor to consider, as patients with idiopathic intracranial hypertension typically receive multiple imaging studies within short periods of time given their consistent recurrence of symptoms (Smith-Bindman et al. 2009). As such, the authors’ institution favored utilization of MR venography over CT venography.

A limitation with pre-operative 3D fusion imaging is that the fusion overlay is static, so there could be slight vessel misregistration caused by deformation of the vessels upon insertion of catheters or wires, potentially requiring additional acquisition to control and adjust registration. Though this workflow can be routinely used in venous sinus stenting procedures, its impact on procedural outcomes (contrast volume, radiation dose, or x-ray time) requires further evaluation in a prospective fashion using a control group.

The main limitation of this case study was its small sample population. There could be also some recall bias for operators performing image assessment. Furthermore, it is difficult to achieve objective ratings regarding the clinical utility of the software given the inherent variability present in providing an overall grade for confidence. Lastly, this study does not provide information about benefits in term of procedural time or radiation doses given the lack of a control arm.

## Conclusion

In summary, the addition of MRV bi-plane 3D fusion during venous sinus stenting procedures for management of IIH is feasible and relatively easy. By providing both consistent visualization and accurate overlay of large cortical veins despite table position alterations, 3D MRV bi-plane guidance may help optimize venous sinus stenting procedural success.

## Data Availability

The datasets used and/or analyzed during the current study are available from the corresponding author on reasonable request.

## Abbreviations

2D: 2 Dimensional
3D: 3 Dimensional
IIH: Idiopathic Intracranial Hypertension
MRV: Magnetic Resonance Venography
